# Fuzzy Temporal Logic Based Railway Passenger Flow Forecast Model

**DOI:** 10.1155/2014/950371

**Published:** 2014-11-05

**Authors:** Fei Dou, Limin Jia, Li Wang, Jie Xu, Yakun Huang

**Affiliations:** ^1^School of Traffic and Transportation, Beijing Jiaotong University, Beijing 100044, China; ^2^Subway Operation Technology Centre, Mass Transit Railway Operation Corporation LTD, Beijing 102208, China; ^3^State Key Laboratory of Rail Traffic Control and Safety, Beijing Jiaotong University, Beijing 100044, China

## Abstract

Passenger flow forecast is of essential importance to the organization of railway transportation and is one of the most important basics for the decision-making on transportation pattern and train operation planning. Passenger flow of high-speed railway features the quasi-periodic variations in a short time and complex nonlinear fluctuation because of existence of many influencing factors. In this study, a fuzzy temporal logic based passenger flow forecast model (FTLPFFM) is presented based on fuzzy logic relationship recognition techniques that predicts the short-term passenger flow for high-speed railway, and the forecast accuracy is also significantly improved. An applied case that uses the real-world data illustrates the precision and accuracy of FTLPFFM. For this applied case, the proposed model performs better than the *k*-nearest neighbor (KNN) and autoregressive integrated moving average (ARIMA) models.

## 1. Introduction

High-speed railway as a kind of large volume passenger transportation mode has been well developed in Europe and Japan and has been developing in China in an even larger scale and has been planned to develop in American continent. In these areas, high-speed railway plays the role of backbone of passenger transportation systems. How to raise operation of the efficiency and how to make the passenger service decision-making more demand-responsive have been the most important focus to the research concerned. As one of the most important basics for the decision-making on high-speed railway transportation pattern and train operation planning, passenger flow forecast is of essential importance, and short-term passenger flow forecast is the key to the success of daily operation management.

Recently, many forecast techniques have been used to solve the prediction problems. Lin and Yang applied the grey forecasting model to forecast the output value of Taiwan's optoelectronics industry accurately from 2000 to 2005 [1]. In [2], four models were developed and tested for the freeway traffic flow forecasting problem. They were the historical average, time-series, neural network, and nonparametric regression models. The nonparametric regression model significantly outperformed the other models. Du and Ren [3] proposed a prediction model of train passenger flow volume to help the railway administration's analysis of running strategies. The model was analysed based on industrial economic indexes and Cobb-Douglas theory to make the prediction. Particularly, ARIMA model has become one of the most common approaches of parametric forecast since the 1970s. The ARIMA model is a linear combination of time-lagged variables and error terms, which has been widely applied in forecasting short-term traffic data such as traffic flow, travel time, and speed. In [4], time series of traffic flow data are characterized by definite periodic cycles. Seasonal autoregressive integrated moving average (ARIMA) and Winters exponential smoothing models were developed. In [5], it was presented that the theoretical basis for modeling univariate traffic condition data streams as seasonal ARIMA process. In [6], Hamed et al. attempted to develop time-series models for forecasting traffic volume in urban arterials, and the Box-Jenkins ARIMA model turned out to be the most adequate model in reproducing all original time series. As stated by Brooks, ARIMA performed well and robustly in modeling linear and stationary time series [7]. However, the applications of ARIMA models were limited because they assumed linear relationships among time-lagged variables and they could not capture the structure of nonlinear relationships [8].

The nonparametric regression models have been applied to forecast transportation demand. However, among these nonparametric techniques, KNN method has been rarely adopted in forecast transportation demand. Robinson and Polak proposed the use of the KNN technique to estimate urban link travel time with single loop inductive loop detector data, and the optimized KNN model was found to provide more accurate estimates than other urban link travel time methods [9].

Neural network model has been frequently adopted to predict. In [10], the time-delay recurrent neural network for temporal correlations and prediction and multiple recurrent neural networks were described. And the best performance is attained by the time-delay recurrent neural network. In [11], a hybrid EMD-BPN forecast approach which combined empirical mode decomposition (EMD) and backpropagation neural networks (BPN) was developed to predict the short-term passenger flow in metro systems. In [12], the forecast model of railway short-term passenger flow based on BP neural network was established based on analyzing the principle of BP neural network and time sequence characteristics of railway passenger flow. In [13], a neural network model was introduced that combines the prediction from single neural network predictors according to an adaptive and heuristic credit assignment algorithm based on the theory of conditional probability and Bayes' rule. In [14], Chen and Grant-Muller reported the application and performance of an alternative neural computing algorithm which involves “sequential or dynamic learning” of the traffic flow process. This indicated the potential suitability of dynamic neural networks with traffic flow data. In [15], Li and Chong-Xin employed chaos theory into forecasting. Delay time and embedding dimension are calculated to reconstruct the phase space and determine the structure of artificial neural network, and the load data of Shanxi province power grid of China is used to show that the model is more effective than classical standard BP neural network model.

Support vector machine technique has also been adopted in forecast. In [16], a modified version of a pattern recognition technique known as support vector machine for regression to forecast the annual average daily traffic was presented. Hu et al. utilized the theory and method of support vector machine regression and established the regressive model based on the least square support vector machine. Through predicting passenger flow on Hangzhou highway in 2000–2008, the authors showed that the regressive model of the least square support vector machine had much higher accuracy and reliability of prediction [17].

Since the problem was introduced, high-speed railway passenger flow forecast is vitally important to the organization of high-speed railway. However, several studies have focused on forecasting short-term high-speed railway passenger flow on the basis of the regularity and randomness of the passenger flow rate. A new method is, therefore, very much needed. Fuzzy temporal logic based passenger flow forecast model (FTLPFFM) is proposed in this paper. Quasi-periodic variation of high-speed railway passenger flow is sufficiently reflected and nonlinear fluctuation of high-speed railway passenger flow is processed using fuzzy logic relationship recognition techniques in the searching process. The proposed model has explicit physical meaning, which reflects variation of high-speed railway passenger flow and has sufficient comprehensibility and interpretability. The characteristics of short-term high-speed railway passenger flow are vitally important to forecast model which is used to improve predictive performance of fuzzy *k*-nearest neighbor by comparing with other predictive methods in short-term high-speed railway passenger flow forecast.

The remainder of this paper is organized as follows. In Section 2, passenger flow characteristics of the high-speed railway and passenger flow variation in adjacent period are summarized. In Section 3, the change degree of passenger flow is divided into eight grades according to cognitive habit and passenger flow change rate is fuzzified. FTLPFFM is proposed in Section 4. In Section 5, the experiment result for the application of FTLPFFM is compared with ARIMA and KNN models when using three statistics: mean absolute error (MAE), mean absolute percentage error (MAPE), and root mean square error (RMSE). And FTLPFFM appears to be more robust and universally fitting. The last section is the conclusion and future work.

## 2. Passenger Flow Feature Extraction

**Figure 1:**
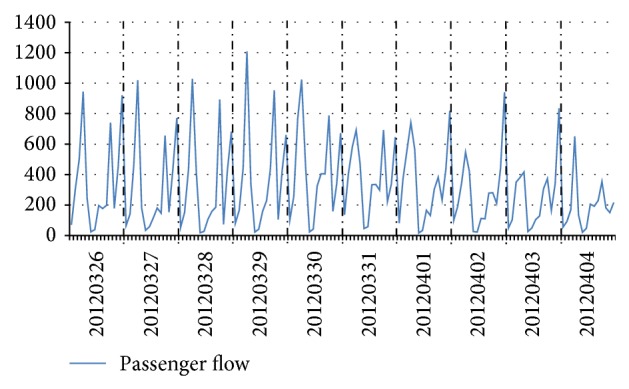
Daily variation of high-speed railway passenger flow.

**Figure 2:**
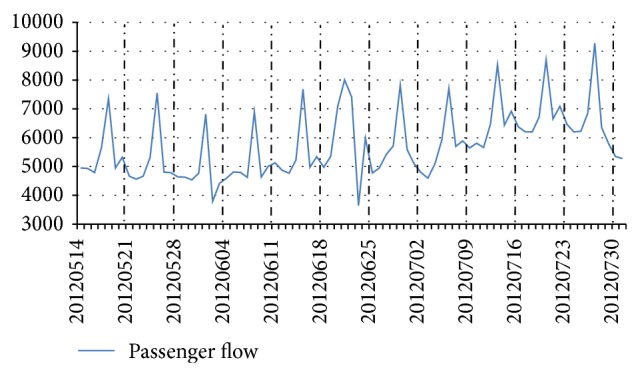
Weekly variation of high-speed railway passenger flow.

In short-term passenger flow forecast, the characteristics of high-speed railway passenger flow are summarized based on time variable because passenger flow has strong correlation to time variable. The data of high-speed railway passenger flow were collected from Beijingnan Railway Station to Jinanxi Railway Station, which is passenger flow in per hour from 26 March to 4 April 2012 (see Figure 1) and daily passenger flow from 14 May to 31 July 2012 (see Figure 2).

Two characteristics of high-speed railway passenger flow are taken into account in FTLPFFM. The first significant characteristic is quasi-periodic which imposes a great impact on passenger flow forecast. The running time of high-speed train is between 6:00 and 24:00 and the passenger flow in morning peak and evening peak is more than other periods, which is revealed in Figure 1. Also, the high-speed railway passenger flow is usually stable from Saturday to Wednesday, increases on Thursday, and reaches the peak on Friday, which is revealed in Figure 2. Therefore, the fluctuation cycle of high-speed railway passenger flow is one day and one week. The second one is nonlinear fluctuation which also imposes a great impact on passenger flow forecast. Specifically, the change rate of passenger flow is instable with nonlinear fluctuation for a short time because of many effect factors, such as passengers' income, travel cost, and service quality of transportation, which is revealed in Figures 1 and 2.

## 3. Regularity of Passenger Flow

Notation:

 
*p*(*t*): the passenger flow in period *t*,
 
*n*: the total number of points of the historical passenger flow series,
 
*p*(*n*): the current passenger flow state,
 
*v*(*t*): the passenger flow change rate from *p*(*t*) to *p*(*t* + 1),
 
*u*_*i*_: the interval of passenger flow change rate,
 
*u*_*i*_′: the intermediate value of *u*_*i*_,  *i* = 1,2,…, 8.

The history passenger flow series is denoted by *p*(1), *p*(2),…, *p*(*t* − 1), *p*(*t*), *p*(*t* + 1),…, *p*(*n* − 1), *p*(*n*). The passenger flow change rates *v*(1), *v*(2),…, *v*(*t* − 1), *v*(*t*), *v*(*t* + 1),…, *v*(*n* − 2), *v*(*n* − 1) between adjacent periods are taken into account, and then the passenger flow change rates are analyzed and variation of passenger flow in adjacent period is summed up.

### 3.1. Change Rate of Passenger Flow

In order to express passenger flow trend in adjacent period clearly and more accurately, passenger flow change rate is normalized.

Define standardized passenger flow change rate *v*(*t*) = (*p*(*t* + 1) − *p*(*t*))/*p*_max⁡_ ∈ [−1,1], and *p*_max⁡_ = max⁡(|*p*(2) − *p*(1)|, |*p*(3) − *p*(2)|,…, |*p*(*n*) − *p*(*n* − 1)|). For *p*(*t* + 1) − *p*(*t*) < 0, the passenger flow descends from period *t* to *t* + 1; for *p*(*t* + 1) − *p*(*t*) > 0, the passenger flow increases from period *t* to *t* + 1; for *p*(*t* + 1) − *p*(*t*) = 0, the passenger flow does not change from period *t* to *t* + 1.

In Table 1, the data are collected from Beijingnan Railway Station to Jinanxi Railway Station in Beijing-Shanghai high-speed railway. For example, the maximum value of the passenger flow change in adjacent periods is calculated as *p*_max⁡_ = max⁡(|*p*(2) − *p*(1)|, |*p*(3) − *p*(2)|,…, |*p*(*n*) − *p*(*n* − 1)|) = 857; the passenger flow change rate from 8:00–8:30 to 8:30–9:00 on October 10th is calculated as *v*(1) = (*p*(2) − *p*(1))/*p*_max⁡_ = (304 − 70)/857 = 0.273. Similarly, we can calculate the passenger flow change rates, which are 0.231, 0.5158, −0.8145, and so forth, as shown in Table 1.

### 3.2. Variation of Passenger Flow

In order to reveal the regularity of the passenger flow trend clearly and express varying degrees of passenger flow change, respectively, we divide passenger flow change rate into eight intervals applying Zadeh's fuzzy set theory [18].

Define the universe of discourse *U* = {*u*_1_, *u*_2_, *u*_3_, *u*_4_, *u*_5_, *u*_6_, *u*_7_, *u*_8_} and partition it into equal length intervals *u*_1_ = [−1, −0.75], *u*_2_ = [−0.75, −0.5], *u*_3_ = [−0.5, −0.25], *u*_4_ = [−0.25,0], *u*_5_ = [0,0.25], *u*_6_ = [0.25,0.5], *u*_7_ = [0.5,0.75], and *u*_8_ = [0.75,1]. The midpoints of these intervals are *u*_1_′ = −0.875, *u*_2_′ = −0.625, *u*_3_′ = −0.375, *u*_4_′ = −0.125, *u*_5_′ = 0.125, *u*_6_′ = 0.375, *u*_7_′ = 0.625, and *u*_8_′ = 0.875. Define fuzzy set *A*_*i*_ based on the redivided intervals; fuzzy set *A*_*i*_ denotes a linguistic value of the passenger flow represented by a fuzzy set, 1 ≤ *i* ≤ 8.

The notations *A*_1_, *A*_2_, *A*_3_, and *A*_4_ denote that passenger flow decrease is too large, larger, microlarge, and less, respectively. Also, the notations *A*_5_, *A*_6_, *A*_7_, and *A*_8_ denote that passenger flow increase is less, microlarge, larger, and too large.

Eight membership functions in this paper sufficiently reflect quasi-periodic variation of high-speed railway passenger flow, and the forecast result of FTLPFFM has better accuracy based on eight membership functions. Define the fuzzy membership function of subset *A*_*i*_, namely,

(1) $$\begin{array}{c} f_{A_{1}}\left(x\right)=\left\{\begin{array}{cc} 1, & -1\leq x\leq -0.75,\\ \frac{-0.5-x}{0.25}, & -0.75<x\leq -0.5,\\ 0, & x>-0.5, \end{array}\right.\\ f_{A_{2}}\left(x\right)=\left\{\begin{array}{cc} \frac{x-\left(-1\right)}{0.25}, & -1<x\leq -0.75,\\ 1, & -0.75<x\leq -0.5,\\ \frac{-0.25-x}{0.25}, & -0.5<x\leq -0.25,\\ 0, & x>-0.25, \end{array}\right.\\ f_{A_{3}}\left(x\right)=\left\{\begin{array}{cc} 0, & x\leq -0.75,\\ \frac{x-\left(-0.75\right)}{0.25}, & -0.75<x\leq -0.5,\\ 1, & -0.5<x\leq -0.25,\\ \frac{-x}{0.25}, & -0.25<x\leq 0,\\ 0, & x>0, \end{array}\right.\\ f_{A_{4}}\left(x\right)=\left\{\begin{array}{cc} 0, & x\leq -0.5,\\ \frac{x-\left(-0.5\right)}{0.25}, & -0.5<x\leq -0.25,\\ 1, & -0.25<x\leq 0,\\ \frac{0.25-x}{0.25}, & 0<x\leq 0.25,\\ 0, & x>0.25, \end{array}\right.\\ f_{A_{5}}\left(x\right)=\left\{\begin{array}{cc} 0, & x\leq -0.25,\\ \frac{x-\left(-0.25\right)}{0.25}, & -0.25<x\leq 0,\\ 1, & 0<x\leq 0.25,\\ \frac{0.5-x}{0.25}, & 0.25<x\leq 0.5,\\ 0, & x>0.5, \end{array}\right.\\ f_{A_{6}}\left(x\right)=\left\{\begin{array}{cc} 0, & x\leq 0,\\ \frac{x}{0.25}, & 0<x\leq 0.25,\\ 1, & 0.25<x\leq 0.5,\\ \frac{0.75-x}{0.25}, & 0.5<x\leq 0.75,\\ 0, & x>0.75, \end{array}\right.\\ f_{A_{7}}\left(x\right)=\left\{\begin{array}{cc} 0, & x\leq 0.25,\\ \frac{x-0.5}{0.25}, & 0.25<x\leq 0.5,\\ 1, & 0.5<x\leq 0.75,\\ \frac{1-x}{0.25}, & 0.75<x\leq 1, \end{array}\right.\\ f_{A_{8}}\left(x\right)=\left\{\begin{array}{cc} 0, & x\leq 0.5,\\ \frac{x-0.5}{0.25}, & 0.5<x\leq 0.75,\\ 1, & 0.75<x<1. \end{array}\right. \end{array}$$

**Figure 3:**
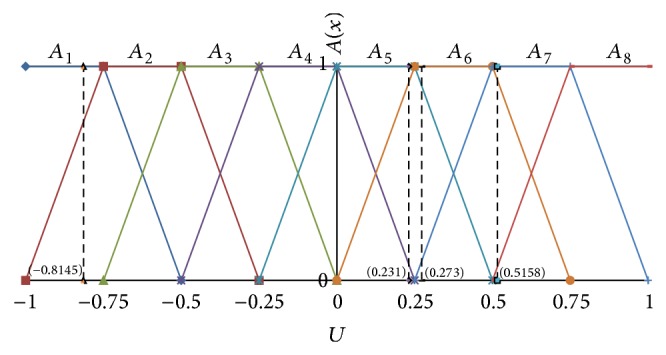
Fuzzified passenger flow change rate.

Different passenger flow change rates can be fuzzified into corresponding fuzzy sets. For example, as seen in Table 1, the passenger flow change rate from 7:00–8:00 to 8:00–9:00 is 0.273, which is fuzzified to *A*_6_. The passenger flow change rate from 8:00–9:00 to 9:00–10:00 is 0.231, which is fuzzified to *A*_5_. The passenger flow change rate from 9:00–10:00 to 10:00–11:00 is 0.5158, which is fuzzified to *A*_7_. And the passenger flow change rate from 10:00–11:00 to 11:00–12:00 is −0.8145, which is fuzzified to *A*_1_. The fuzzification process is depicted in Figure 3. Some fuzzified passenger flow change rates are listed in Table 1.

Fuzzy logic relationships are established by putting two consecutive fuzzy sets, as follows:

(2) $$\begin{array}{c} A_{j}⟶A_{p},A_{p}⟶A_{q},\ldots ,A_{s}⟶A_{t}. \end{array}$$

“*A*_*j*_ → *A*_*p*_” denotes that “the fuzzified passenger flow change rate is *A*_*j*_ from period *t* − 1 to *t* and then the fuzzified passenger flow change rate is *A*_*p*_ from period *t* to *t* + 1”.

**Figure 4:**
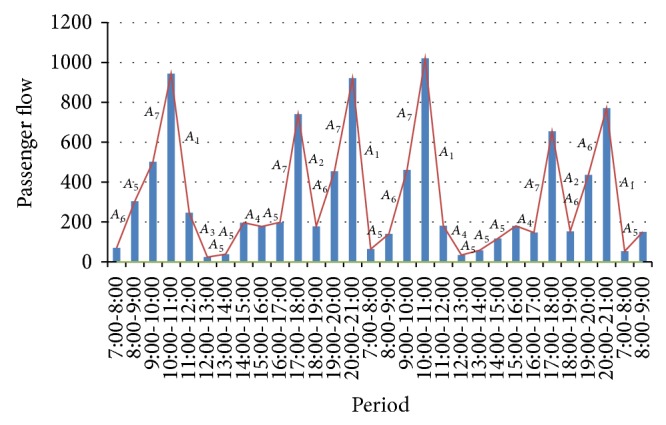
Passenger flow change rate relationships.

As seen in Figure 4, the fuzzified passenger flow change rate from 7:00–8:00 to 8:00–9:00 is *A*_6_ and from 8:00–9:00 to 9:00–10:00 is *A*_5_. Hence, we can establish an fuzzy logic relationship as *A*_6_ → *A*_5_. Likewise, from Table 1, we can establish the fuzzy logic relationships as *A*_6_ → *A*_5_, *A*_5_ → *A*_7_, *A*_7_ → *A*_1_, *A*_1_ → *A*_3_, and so forth. Some fuzzy logic relationships are listed in Table 2.

## 4. Fuzzy Temporal Logic Based Passenger Flow Forecast Model

Notation:

 
*k*_*i*_: the number of the passenger flow change rate belonging to *A*_*i*_,
 
*k*: the size of neighborhood,
 
*d*: the dimension of the current passenger flow change rate vector.

### 4.1. K-Nearest Neighbor Model

The *K*-nearest neighbor model is one of the most famous pattern recognition statistical models. The KNN model defines neighborhoods as those *k* cases with the least distance to the input state [19]. The literature indicates that Euclidean distance is usually used to determine the distance between the input state and cases in the database [20]. The predictions can be calculated by averaging the observed output values for cases that fall within the neighborhood when the neighborhood is obtained.

For example, a passenger flow series *p*(1), *p*(2),…, *p*(*t* − 1), *p*(*t*), *p*(*t* + 1),…, *p*(*n* − 1), *p*(*n*) where *n* is the total number of points of the series. We search the series to find the nearest neighbors, of the current state *p*(*n*). Then, we predict *p*(*n* + 1) on the basis of those nearest values; for example, if the neighborhood size was *k* = 1 and the nearest passenger flow was *p*(*t*), then we would predict *p*(*n* + 1) on the basis of *p*(*t* + 1). The value of *k* in KNN model is more often obtained by empirical analysis. In general, the steps of the KNN model can be listed as follows.

**Step 1 .**

Identify the neighborhood size *k* and the original state of variables.

**Step 2 .**

Input all original state of variables into the development database.

**Step 3 .**

Calculate Euclidean distance of the current state of variables to each state in development database.

**Step 4 .**

Choose output of *k*-nearest neighborhood on the basis of *k* shortest Euclidean distance from development database.

**Step 5 .**

Calculate the predictive value which is the average of the output of *k*-nearest neighborhood.

### 4.2. Fuzzy Temporal Logic Based Passenger Flow Forecast Model

Suppose *P*(*t*) = [*p*(*t*), *p*(*t* + 1),…, *p*(*t* + *d* − 1), *p*(*t* + *d*)] is the *t*-period historical passenger flow state vector and *V*(*t*) = [*v*(*t*), *v*(*t* + 1),…, *v*(*t* + *d* − 2), *v*(*t* + *d* − 1)] is the historical passenger flow change rate vector. For *t* = *n* − *d*, *P*(*n* − *d*) and *V*(*n* − *d*) are the current passenger flow state vector and the current passenger flow change rate vector.

#### 4.2.1. Distance Metric

Give the state matrix of passenger flow and the matrix of the passenger flow change rate so as to compare the relationship among the different periods of passenger flow more clearly. The state matrix of passenger flow is given by

(3) P1P2⋮Pt−d⋮Pn−d  =p1p2⋯p1+dp2p3⋯p2+d⋮⋮⋮pt−dpt−d+1⋯pt⋮⋮⋮pn−dpn−d+1⋯pn.

The matrix of the passenger flow change rate is given by

(4) V1V2⋮Vt−d⋮Vn−d  =v1v2⋯vdv2v3⋯v1+d⋮⋮⋮vt−dvt−d+1⋯vt−1⋮⋮⋮vn−dvn−d+1⋯vn−1.

A common approach to measure the “nearness” in KNN model is to use the Euclidean distance [18]. Therefore, the Euclidean distances of the passenger flow state vectors and the passenger flow change rate vectors are as follows:

(5) d1=Pn−d−Pt−d=∑j=0dpn−j−pt−j2,

(6) d2=Vn−d−Vt−d=∑j=1dvn−j−vt−j2.

#### 4.2.2. Forecast Passenger Flow Change Rate

Suppose the neighborhood search procedure identifies *k* neighbors, the passenger flow state vectors of the *k* neighbors are *P*(*t*′ − *d*, *h*) = [*p*(*t*′ − *d*, *h*), *p*(*t*′ − *d* + 1, *h*),…, *p*(*t*′ − 1, *h*), *p*(*t*′, *h*)] (*h* = 1,2,…, *k*) and *p*(*t*′ + 1, *h*) is next to *p*(*t*′, *h*), and *V*(*n* − *d*) = [*v*(*n* − *d*), *v*(*n* − *d* + 1),…, *v*(*n* − 1)] is the current passenger flow state vector. The passenger flow change rates corresponding to *p*(*t*′ + 1, *h*) and *p*(*t*′, *h*) are *v*(*t*′, *h*) = (*p*(*t*′ + 1, *h*) − *p*(*t*′, *h*))/*p*_max⁡_, *h* = 1,2,…, *k*. The number of the passenger flow change rate *v*(*t*′, *h*) belonging to *A*_*i*_ is *k*_*i*_, and the value of *v*(*t*′, *h*) corresponding to *A*_*i*_ is *u*_*i*_′.

An approach to forecasting is to compute an average of *v*(*t*′, *h*)s of the neighbors that have fallen within the neighborhood:

(7) v(n)=k1u1′+k2u2′+k3u3′+k4u4′+k5u5′+k6u6′+k7u7′+k8u8′∑i=18ki.

#### 4.2.3. Steps of FTLPFFM

The establishment of FTLPFFM is based on fuzzy *k*-nearest neighbor prediction method.

Steps of FTLPFFM are as follows.

**Step 1 .**

Start with a minimal neighborhood size, *k* = 1.

**Step 2 .**

Start with a minimal dimension of the current passenger flow change rate vector, *d* = 1.

**Step 3 .**

Start with period *l* = *n* + 1 to predict passenger flow.

**Step 4 (match to find the elementary neighbors).**

Find the nearest matches for the current passenger flow state vector *P*(*l* − *d* − 1) = [*p*(*l* − *d* − 1), *p*(*l* − *d*),…, *p*(*l* − 2), *p*(*l* − 1)] by searching the passenger flow series *p*(1), *p*(2),…, *p*(*n* − 1) using (5), and then sort them in ascending order. Suppose an index *t*′ − *d*, for which the nearest matching passenger flow state vector is *P*(*t*′ − *d*) = [*p*(*t*′ − *d*), *p*(*t*′ − *d* + 1),…, *p*(*t*′ − 1), *p*(*t*′)] and the historical passenger flow change rate vector associated is *V*(*t*′ − *d*) = [*v*(*t*′ − *d*), *v*(*t*′ − *d* + 1),…, *v*(*t*′ − 2), *v*(*t*′ − 1)]. Here, the current passenger flow change rate vector is *V*(*l* − *d* − 1) = [*v*(*l* − *d* − 1), *v*(*l* − *d*),…, *v*(*l* − 3), *v*(*l* − 2)]; search the same fuzzy logical relationships *A*_*i*_′ → *A*_*j*_′ → ⋯→*A*_*p*_′ → *A*_*q*_′ for *V*(*t*′ − *d*) and *A*_*i*_ → *A*_*j*_ → ⋯→*A*_*p*_ → *A*_*q*_ for *V*(*l* − *d* − 1), and choose the top 2*k* matches which are the elementary neighbors. The appropriate passenger flow change rate vectors of 2*k* will be discussed below.

**Step 5 (match to find the nearest neighbors).**

Find the nearest matches for *V*(*l* − *d* − 1) by searching all the historical passenger flow change rate vectors *V*(*t*′ − *d*) using (6), and then sort them in ascending order and choose the top *k* matches. They are the nearest neighbor passenger flow state vectors *P*(*t*′ − *d*, *h*) = [*p*(*t*′ − *d*, *h*), *p*(*t*′ − *d* + 1, *h*),…, *p*(*t*′ − 1, *h*), *p*(*t*′, *h*)], and output *p*(*t*′, *h*) and *p*(*t*′ + 1, *h*), *h* = 1,2,…, *k*.

**Step 6 .**

Estimate the passenger flow change rate *v*(*l* − 1) using (7).

**Step 7 .**

Calculate predictive value of passenger flow $\overset{-}{p}(l)=p(l-1)+p_{\max}\cdot v(l-1)$ and add it to the database; repeat Step 4 to Step 7 with regard to *l* = *l* + 1 until *l* = *M*, *M* is the last period.

**Step 8 .**

Calculate RMSE between the actual values and predicted values, which is given by

(8) $$\begin{array}{c} \text{RMSE}=\sqrt{\frac{1}{M-n}\overset{M}{\underset{i=n+1}{\sum }}{\left[\overset{-}{p}\left(i\right)-p\left(i\right)\right]}^{2}}, \end{array}$$

where $\overset{-}{p}(i)$ is the predicted value of actual value *p*(*i*).

**Step 9 .**

Repeat Steps 3 to 8 for vector dimensions of *d* + 1, *d* + 2,…, *d*_max⁡_.

**Step 10 .**

Repeat Steps 2 to 9 for neighborhood sizes of *k* + 1, *k* + 2,…, *k*_max⁡_.

**Step 11 .**

Choose the optimal predictive values of passenger flow which yields minimal RMSE by optimizing the vector dimensions and the neighborhood.

Choose the maximum dimension of the current passenger flow change rate vector and the maximum neighborhood size according to the characteristics of the passenger flow. Smith and Demetsky (1994) [20] found that the best predictions were generated using *k* = 10, and Karlsson and Yakowitz (1987) [21] proposed that the best forecast values were generated using *k* = 3. Wang et al. (2011) [22] and Oswald et al. (2001) [23] revealed that the best results were obtained when *k* ≤ 30. We obtain the best predicted values of passenger flow as nearly all fall within the search space, which is 1 ≤ *k* ≤ 30 and 1 ≤ *d* ≤ 20, by numerous experiments using different dataset.

## 5. Case Study

The data were obtained from National Key Technology Research and Development Program, State Key Laboratory of Rail Traffic Control and Safety, Beijing Jiaotong University. The database was per hour passenger flow between 7:00 and 21:00 from Beijing to Jinan in Beijing-Shanghai high-speed railway, which was split into two parts separately: an estimation data set and a test data set. The estimation data set was collected from 1 July to 31 December 2011 (2576 observations) and the test data set was collected from 1 to 22 January 2012 (300 observations).

**Figure 5:**
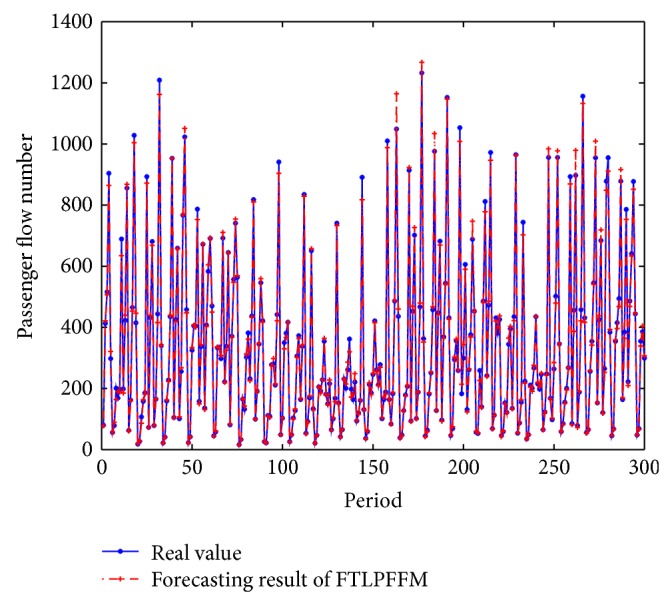
Comparisons of predictive values and real values.

According to the passenger flow characteristics, we can set *d*_max⁡_ = 10 and *k*_max⁡_ = 20. The developed model for the passenger flow of the high-speed railway was implemented using MATLAB version 7.1. The best results were obtained when *k* = 10 and *d* = 4, which can be seen from RMSE performance, and RMSE = 2.7046. The best prediction results and actual values are shown in Figure 5.

ARIMA model is a benchmarking method in forecasting field, but it is a gray box model, which cannot reflect the underlying structural properties. KNN model has dynamic adaptability to the data which is a white box model and has sufficient comprehensibility. And FTLPFFM is presented based on KNN forecasting model and has sufficient comprehensibility and interpretability. Therefore, FTLPFFM is compared with ARIMA and KNN models using three statistics: MAE, MAPE, and RMSE, as is shown in Table 3. And (9) shows how MAE and MAPE are computed, respectively. Consider

(9) $$\begin{array}{c} \text{MAE}=\frac{1}{M-n}\overset{M}{\underset{i=n+1}{\sum }}\left|\overset{-}{p}\left(i\right)-p\left(i\right)\right|,\\ \text{MAPE}=\frac{1}{M-n}\overset{M}{\underset{i=n+1}{\sum }}\frac{\left|\overset{-}{p}\left(i\right)-p\left(i\right)\right|}{p\left(i\right)}. \end{array}$$

**Figure 6:**
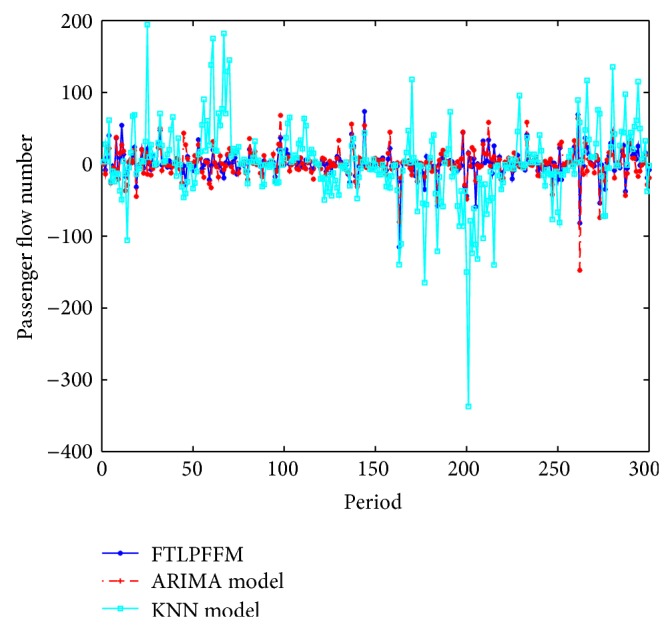
The absolute error of three models.

**Figure 7:**
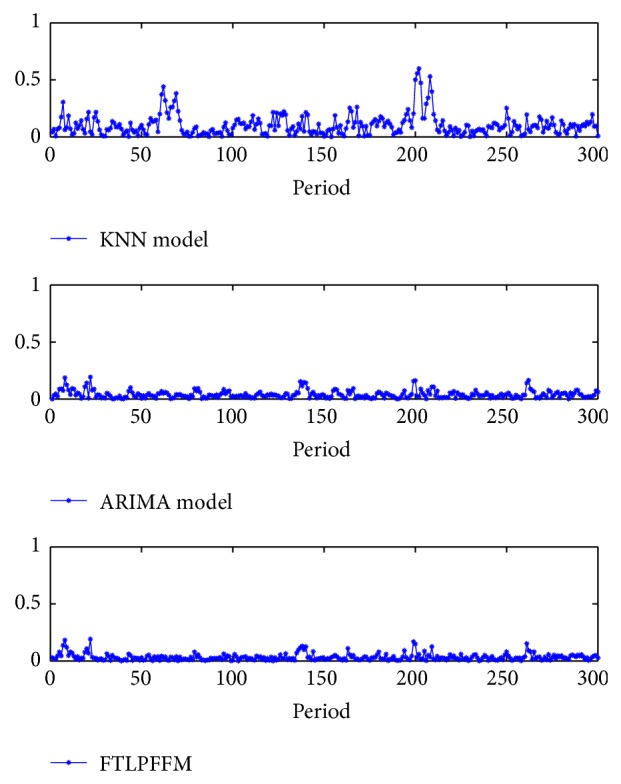
The absolute relative deviation of three models.

The absolute error and the absolute relative deviation of three models are computed as shown in Figures 6 and 7.

The result of the comparison between the prediction results and actual values indicates that the proposed model has been shown to be effective and the error is acceptable.

## 6. Conclusion and Future Work

Railway transport is an increasingly popular transportation mode for medium-long distance journey in many countries in recent years. The short-term passenger flow forecast has played a key role in high-speed railway intelligent transportation system. In this paper, a FTLPFFM is developed to measure uncertainty of high-speed railway passenger flow for railway passenger transport management. In FTLPFFM, the past sequences of passenger flow are considered to predict the future passenger flow using fuzzy logic relationship recognition techniques in the searching process. The results reveal that the forecast accuracy (measured with MAE, MAPE, and RMSE) of the FTLPFFM was significantly better than the accuracy levels of the ARIMA and KNN models. Fuzzy temporal logic based passenger flow forecast model also provides a theoretical foundation in decision-making of resource allocation. In a more general sense of application, the proposed method could be adapted in multimodal transportation systems especially in railway transport and metro transport. For future work, one possible extension of this research is to improve forecast accuracy via properly applying data fusion and pattern recognition techniques.

## Figures and Tables

**Table 1:** The value of passenger flow, passenger flow change degree, passenger flow change rate, and fuzzy set.

Date	Period	Passenger flow	The value of passenger flow change degree	Passenger flow change rate	Fuzzy set
2011.10.10	7:00–8:00	70	234	0.273	*A*_6_
	8:00–9:00	304	198	0.231	*A*_5_
	9:00–10:00	502	442	0.5158	*A*_7_
	10:00–11:00	944	−698	−0.8145	*A*_1_
	11:00–12:00	246	−222	−0.259	*A*_3_
	12:00–13:00	24	14	0.0163	*A*_5_
	13:00–14:00	38	158	0.1844	*A*_5_
	14:00–15:00	196	−19	−0.0222	*A*_4_
	15:00–16:00	177	21	0.0245	*A*_5_
	16:00–17:00	198	543	0.6336	*A*_7_
	17:00–18:00	741	−563	−0.6569	*A*_2_
	18:00–19:00	178	276	0.3221	*A*_6_
	19:00–20:00	454	467	0.5449	*A*_7_
	20:00–21:00	921	−857	−1	*A*_1_

2011.10.11	7:00–8:00	64	76	0.0887	*A*_5_
	8:00–9:00	140	321	0.3746	*A*_6_
	9:00–10:00	461	559	0.6523	*A*_7_
	10:00–11:00	1020	−839	−0.979	*A*_1_
	11:00–12:00	181	−147	−0.1715	*A*_4_
	12:00–13:00	34	24	0.028	*A*_5_
	13:00–14:00	58	59	0.0688	*A*_5_
	14:00–15:00	117	63	0.0735	*A*_5_
	15:00–16:00	180	−33	−0.0385	*A*_4_
	16:00–17:00	147	508	0.5928	*A*_7_
	17:00–18:00	655	−502	−0.5858	*A*_2_
	18:00–19:00	153	283	0.3302	*A*_6_
	19:00–20:00	436	335	0.3909	*A*_6_
	20:00–21:00	771	−718	−0.8378	*A*_1_

2011.10.12	7:00–8:00	53	97	0.1132	*A*_5_
	8:00–9:00	150	282	0.3291	*A*_6_
	9:00–10:00	432	—	—	—

**Table 2:** The fuzzy logic relationship of fuzzified passenger flow change rate.

*A*_6_ → *A*_5_,	*A*_5_ → *A*_7_,	*A*_7_ → *A*_1_,	*A*_1_ → *A*_3_,	*A*_3_ → *A*_5_,
*A*_5_ → *A*_5_,	*A*_5_ → *A*_4_,	*A*_4_ → *A*_5_,	*A*_5_ → *A*_7_,	*A*_7_ → *A*_2_,
*A*_2_ → *A*_6_,	*A*_6_ → *A*_7_,	*A*_7_ → *A*_1_,	*A*_1_ → *A*_5_,	*A*_5_ → *A*_6_,
*A*_6_ → *A*_7_,	*A*_7_ → *A*_1_,	*A*_1_ → *A*_4_,	*A*_4_ → *A*_5_,	*A*_5_ → *A*_5_
*A*_5_ → *A*_5_,	*A*_5_ → *A*_4_,	*A*_4_ → *A*_7_,	*A*_7_ → *A*_2_,	*A*_2_ → *A*_6_,
*A*_6_ → *A*_6_,	*A*_6_ → *A*_1_,	*A*_1_ → *A*_5_,	—	—

**Table 3:** The comparison between ARIMA, KNN, and FTLPFFM.

Prediction model	MAE	MAPE	RMSE
KNN	5.4623	0.0453	3.5684
ARIMA	2.9642	0.0401	3.1812
FTLPFFM	1.9117	0.0328	2.7046
